# Dielectric, Thermal and Mechanical Properties of l,d-Poly(Lactic Acid) Modified by 4′-Pentyl-4-Biphenylcarbonitrile and Single Walled Carbon Nanotube

**DOI:** 10.3390/polym11111867

**Published:** 2019-11-12

**Authors:** Patryk Fryń, Krzysztof Artur Bogdanowicz, Piotr Krysiak, Monika Marzec, Agnieszka Iwan, Adam Januszko

**Affiliations:** 1 Institute of Physics, Jagiellonian University, Lojasiewicza 11, 30-348 Krakow, Poland; patryk.fryn@doctoral.uj.edu.pl; 2 Military Institute of Engineer Technology, Obornicka 136 Str., 50-961 Wroclaw, Poland; bogdanowicz@witi.wroc.pl (K.A.B.); krysiak@witi.wroc.pl (P.K.); 3 Faculty of Security and Safety Research, General Tadeusz Kosciuszko Military University of Land Forces, Czajkowskiego 109 Str., 51-147 Wroclaw, Poland; adam.januszko@awl.edu.pl

**Keywords:** l,d-poly(lactic acid), 5CB, SWCN, hybrid materials, electric properties, mechanical properties, thermographic camera

## Abstract

We report here the preparation and thermal, electrical and mechanical characterization of binary and ternary films based on l,d-poly(lactic acid) (l,d-PLA) and 4′-pentyl-4-biphenylcarbonitrile (5CB) and Single Walled Carbon Nanotubes (SWCN) with various weight ratio. The transitions for all investigated hybrid compositions detected by differential scanning calorimetry method were shifted to lower temperatures with increasing the concentration of 5CB in the mixture with polymer. Frequency domain dielectric spectroscopy method and thermal imaging together with polarized optical microscope were used to study electric and structural properties of created hybrid compositions. The best electrical conductivity was observed for hybrid composite l,d-PLA:5CB:SWCN with ratio 10:1:0.5 *w*/*w*/*w* - resistance of 41.0 Ω and thermal response up to 160 °C without causing any damages. Films in crystal form are much more inflexible than in amorphous and can be explain by the cold crystallization occurs at heating while the materials changed their physical state. The value of ε′ increases with increasing the 5CB admixture. Moreover, the addition of 5CB to l,d-PLA resulted in increased flexibility of polymeric base films. The best material flexibility and short-term strength were obtained for l,d-PLA sample with 9% 5CB content.

## 1. Introduction

Hybrid films based on polymer and liquid crystalline materials are at the present time wide experimentally and theoretically investigated towards use in various of flexible devices [1,2,3,4,5,6,7,8,9,10,11,12]. Films and fibres are made with an attempt to build light emitting diodes, transistors or solar cells [13,14,15,16,17,18,19,20,21]. However, main disadvantages of the binary and ternary films are the fact that additives have negative impact on the physical characteristics of flexible polymers and reducing their flexibility and transparency. Various technological concepts are proposed towards created films with good mechanical properties. For example, Buyuktanir et al. [22] prepared and investigated hybrid fibres based on l,d-PLA and 4′-pentyl-4-biphenylcarbonitrile (5CB) obtained by electrospinning. Well known and a widely used liquid crystal 5CB, exhibiting a transition from crystalline to liquid crystalline nematic phase at 24 °C and from nematic to isotropic one at 35 °C [23].

Serrano et al. [24] investigated phase behaviour of a binary liquid mixture composed of methanol and 5CB. Hori et al. [25] analysed phase behaviour and dynamics in blends of 5CB and polystyrene by using dielectric spectroscopy. On the other hand, Javadian et al. [26] investigated non-covalent intermolecular interactions of 5CB doped with graphene oxide.

In our previous study we created binary hybrid thin films based on Single-Walled Carbon Nanotubes (SWCN) and l,d-PLA [27]. Our study showed that a 0.01% SWCN concentration gave translucent supports; however, it did not exhibit sufficient conductivity. An increase in the amount of SWCN to 5% was accompanied by an increase in conductivity to about 1.01 × 103 S/m; however, the films were not fully translucent.

Here, in this article, we are particularly interested in analysing the impact of 4′-pentyl-4-biphenylcarbonitrile (5CB) on the thermal, dielectric and mechanical properties of l,d-poly(lactic acid) via increase the amount of 5CB in the polymer matrix from 0 to 50%. Moreover, we fabricated and analysed flexible, semiconducting ternary film based on l,d-PLA:5CB:SWCN composition with weight ratio of SWCN from 0.01 to 0.5 for possible application as flexible electrode in organic devices.

Investigated in this work biodegradable l,d-PLA is still very attractive for the science and industry bearing in mind facts such as: (i) it is an environmentally friendly polymer (compostable and it derives from renewable resources) and (ii) it shows a high elastic modulus and a high transparency [28,29]. However, among disadvantages of l,d-PLA the most important ones are low glass transition temperature, low thermal stability, high brittleness and low crystallization rate. Binary and ternary mixtures or blends were created to improved selected properties of l,d-PLA and examples of some components are listed:

(i) a low molecular weight plasticizer, for example, acetyl tributyl citrate [30,31];
(ii) polymers such as: poly(butylene adipate-co-terephthalate) [32,33] poly(butylene succinate) [34,35], poly(hydroxy butyrate) [36,37], polyaniline with multiwalled carbon nanotubes as flexible free-standing electrode for supercapacitors [38];
(iii) carbon nanotubes [39,40,41,42,43,44,45];
(iv) graphene and graphene oxide [46,47,48];
(v) liquid crystalline poly [4,4′-bis(6-hydroxyhexyloxy) biphenyl phenylsuccinate] as functional chain to copolymerize with PLA, towards improve the flexibility of PLA and caused interactions with multiwalled carbon nanotubes via π–π interaction [49];
(vi) magnesium as filament based 3D printing [50];
(vii) TiO_2_ for antibacterial packaging [51];
(viii) ZnO as potential antimicrobial food packaging materials [52];
(ix) high-density polyethylene/carbon black composites as electrically conductive composites with a low percolation threshold [53].

The aim of this work was to increase the flexibility and electrical conductivity of l,d-PLA by addition of SWCN and 5CB. Moreover, we wanted to modulate better properties of l,d-PLA by including morphological characteristic of the low molecular weight liquid crystalline 5CB compound. SWCN have extremely good mechanical and electrical properties due to their strong sp^2^ carbon-carbon bondings and their geometric arrangement, therefore they are ideal reinforcement fillers for l,d-PLA based composites. In this work we proposed to improve selected limitation of an environmentally friendly polymer, such as l,d-PLA, by adding 5CB and SWCN towards reduce high brittleness and improve electrical properties.

The observed changes in the thermal (differential scanning calorimetry, polarizing optical microscopy), electrical (thermographic camera, dielectric experiments) and mechanical properties of the investigated binary and ternary hybrids based on l,d-PLA, 5CB and SWCN suggested that they depend on the additives weight ratio ordering the polymer matrix.

## 2. Experimental

### 2.1. Materials

The 4′-pentyl-4-biphenylcarbonitrile (5CB) and Single Walled Carbon Nanotubes (SWCN) were used as received from Sigma-Aldrich (Saint Louis, MO, USA). SWCN used have average diameter 0.84 nm, median length 1 µm, ≥ 95% carbon basis (≥ 99% as carbon nanotubes). l,d-PLA was used as received from GALACTIC (Brussels, Belgium).

#### 2.1.1. Preparation of l,d-PLA:5CB Hybrid Materials

**Figure 1:**
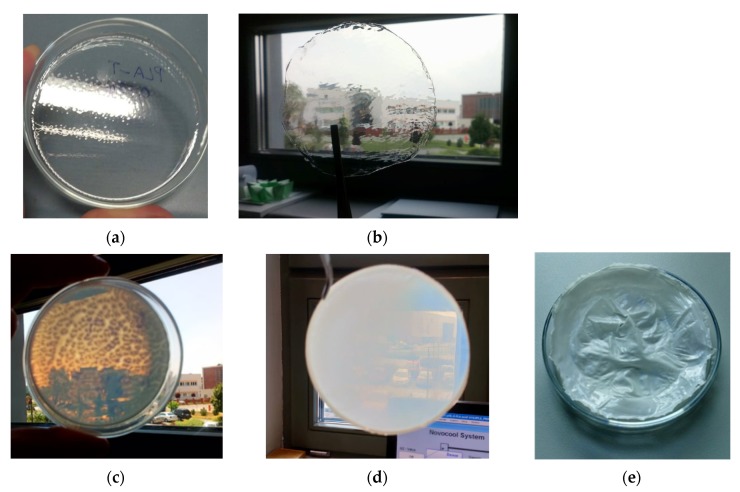
Photos of films of pure l,d-PLA (**a**) and composites l,d-PLA:5CB for different concentrations: 10: 1 *w*/*w* (**b**), 10:3 *w*/*w* (**c**), 10:5 *w*/*w* (**d**) and 10:10 *w*/*w* (**e**); the diameter of all created films is 8 cm.

All fabricated hybrid layers with different concentration of l,d-PLA:5CB were prepared in the same way. l,d-PLA was mixed with appropriate amount of 5CB and poured 4 mL chloroform. The solution was mixed on the magnetic stirrer for at least 2 h and next it was sonicated for 40 min. Subsequent mixture was drop-casted on the glass substrate of 8 cm diameter to create flexible films after chloroform evaporation (see Figure 1). The thickness of the hybrid films was determined by profilometer and were found in the range of 48 ± 4 to 89 ± 4 μm.

#### 2.1.2. Preparation of l,d-PLA:5CB:SWCN Hybrid Materials

**Figure 2:**
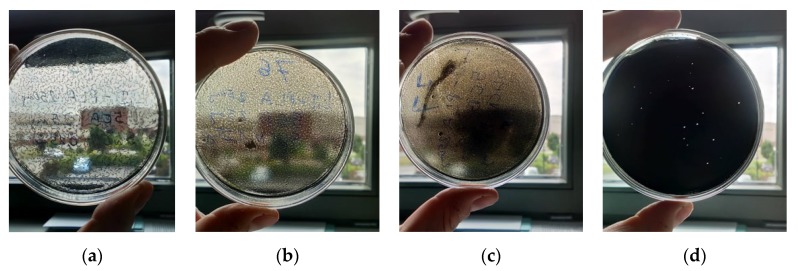
Photos of films of composites l,d-PLA:5CB:SWCN for different ratio: 10:1:0.01 *w*/*w*/*w* (**a**), 10:1:0.05 *w*/*w*/*w* (**b**), 10:1:0.1 *w*/*w*/*w* (**c**), 10:1:0.5 *w*/*w*/*w* (**d**) the diameter of all created films is 8 cm.

All created hybrid layers with different concentration of l,d-PLA:5CB:SWCN were prepared in the same way. l,d-PLA and 5CB with weight ratio 10:1 was mixed with proper amount of SWCN and poured chloroform. The further procedure was as in the case of preparation of l,d-PLA:5CB hybrid layers. The created layers are presented in Figure 2. The thickness of the hybrid films was determined by profilometer (Bruker Co., Billerica, MA, USA) and were found in the range of 43 ± 2 to 55 ± 2 μm.

### 2.2. Characterization of Methods

Differential scanning calorimetry (DSC) measurements were done by using Pyris 1 Perkin Elmer calorimeter (PerkinElmer, Waltham, MA, USA) to find transition temperatures of the new created hybrid materials. The samples of ca. 10 mg were placed into 30 μL aluminium crucibles and tightly closed with a press. The measurements were performed with 5 °C/min rate both on heating and cooling. At least three cycles of heating and cooling were done for each sample to ensure repeatability of the results.

Texture observations were performed using Nikon Eclipse LV100POL polarizing microscope (NIKON Inc., Tokyo, Japan) equipped with the Fine Instruments WTMS–14C heating stage. The images were registered by the computer-controlled camera Canon EOS 600D. A small piece of each created layer (c.a. 1 cm^2^) was placed between the glass substrate and the cover glass and put inside the heating stage.

Frequency domain dielectric spectroscopy (FDDS) method was used to study electric properties of new created hybrid layers. For this purpose, the Alpha analyser (Novocontrol Technologies GmBbH & Co. KG, Montabaur, Germany) Novocontrol Technologies equipped with an automatically controlled cryostat Novocool was used. Measurements were performed in the plate capacitor geometry, in the frequency range from 0.5 Hz to 2.5 MHz versus temperature with temperature step equal to 1 °C. Thin round pastilles (diameter 20 mm, thickness of c.a. 50–100 μm) were cut off from the created layers and placed between two electrodes. Measurement of pure 5CB was done in the cell with golden electrodes of 25 mm^2^ area and thickness c.a. 5 μm (AWAT Company, Warsaw, Poland).

The thickness of created hybrid layers was determined using DektakXT (Bruker Co., Billerica, MA, USA) Stylus profilometer working in N-Lite mode which ensures a very low stylus load (radius 2 µm, load 0.3 mg) on sample surface assuring non-destructive way of analysis. Layers were placed at glass substrate and profiles of cross section were measured. Profiles were edited by establish the ground level which was set for glass substrate. Thickness was determinate by the difference between the average profile height and the substrate level.

Analysis of the optical textures was done by Fiji program (The Laboratory for Optical and Computational Instrumentation, Madison, WI, USA) which is distribution of ImageJ [54]. Each texture image was converted on 8-bit type. Brightness contrast was adjusted by auto function. The background was removed with rolling ball radius 150 pixels. The threshold was settled by Shanbhag algorithm [55]. Next images were process by dilate operations. Analysed particles were settled from 2 μm^2^ up to infinity to cut off the single pixel’s contributions.

Tensile mechanical properties were measured using a testing machine Instron 33R4469 (Instron, Norwood, MA, USA) with a load cell of 5 kN with software Bluehill 3.0 (Instron, Norwood, MA, USA). Samples were in a form of thin strips and uniform width of 12 mm. Samples were tested at room temperature at a crosshead speed of 10 mm/min.

Thermal behaviour was performed as described in our previous work [56]. Thermal images were collected using a thermographic camera (VIGOcam v50, VIGO System S.A, Ożarów Mazowiecki, Poland) while bias voltage between 0 and 10 V was applied using a multichannel potentiostat-galvanostat (PGStat Autolab M101, Metrohm, Barendrecht, Nederland), connected to computer.

## 3. Results and Discussion

The l,d-PLA in this work was mixed with well-known organic compound exhibiting nematic phase in the room temperature—4′-pentyl-4-biphenylcarbonitrile (5CB), in different ratio and drop-casted on glass substrate to create flexible film after chloroform evaporation. In Figure 1a–e photos of created films along with increase the amount of 5CB are presented. The transparency of created film decreased with increasing the 5CB admixture. In details, films without and with very small content of 5CB (10:1 *w*/*w*) were transparent (Figure 1a,b), while films with a higher 5CB admixture (10:3 *w*/*w* and 10:5 *w*/*w*) were partially transparent (Figure 1c,d). Moreover, thin films with the ratio of l,d-PLA to 5CB equal to 10:10 *w*/*w* were not transparent (Figure 1e) and the film attached very strongly to the glass, than it was hard to peel off from the substrate without causing damage. It seems that the stronger adhesion is for the surface of the thin film with the ratio l,d-PLA to 5CB 10:10 *w*/*w* as compare with other created films due to the high viscosity of 5CB.Therefore, compositions with a higher than 10:10 *w*/*w* 5CB admixture were not prepared.

### 3.1. DSC Measurements and Texture Observations Before and After Mechanical Deformation

**Figure 3:**
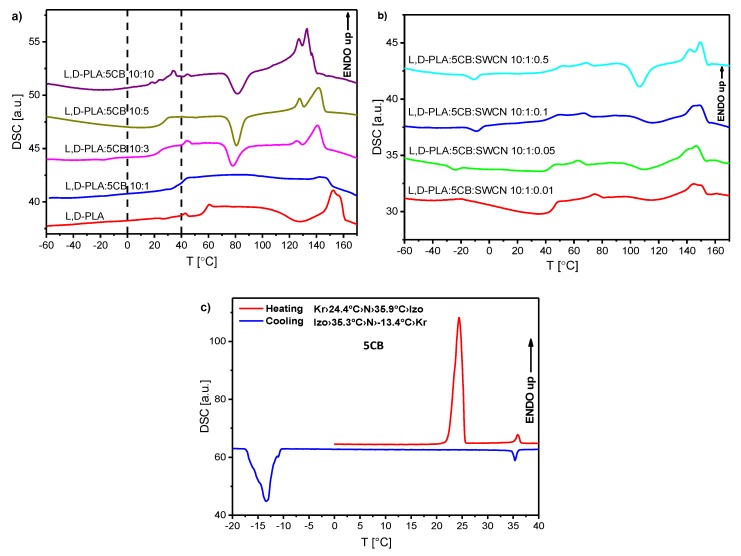
DSC curves for binary (**a**) and ternary (**b**) mixtures registered during heating and for pure 5CB during heating and cooling (**c**).

l,d-PLA and its mixtures with 5CB as well as l,d-PLA and its mixtures with 5CB and SWCN for different concentrations were studied using DSC method to determine the influence of content of 5CB and SWCN on the thermal behaviour. As it is seen in Figure 3 and Table 1, all the transitions are shifted to lower temperatures with increasing the concentration of 5CB in the mixture, which is connected with low transition temperatures of pure 5CB: the liquid crystalline nematic phase exists between 24.4 and 35.9 °C (see Figure 3c). On the other hand, addition of SWCN does not influence much on the transition temperatures (Figure 3b, Table 1).

Only for composition 10:10 *w*/*w* there were visible additional peaks which not appear on DSC curve for pure l,d-PLA. According to the literature [57] the peak registered on heating at ca. 150 °C is related to melting of l,d-PLA, while around 55 °C to glass-transition [58]. 

**Figure 4:**
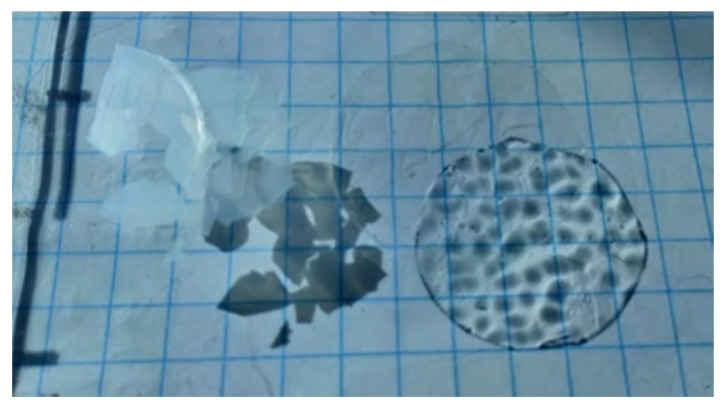
Shadow of broken parts of pure l,d-PLA film in crystalline (**on the left**) and amorphous (**on the right**) phase.

Above the glass transition the cold crystallization occurred and properties of compositions changed—they become fragile and break easily, as it is presented in Figure 4.

**Figure 5:**
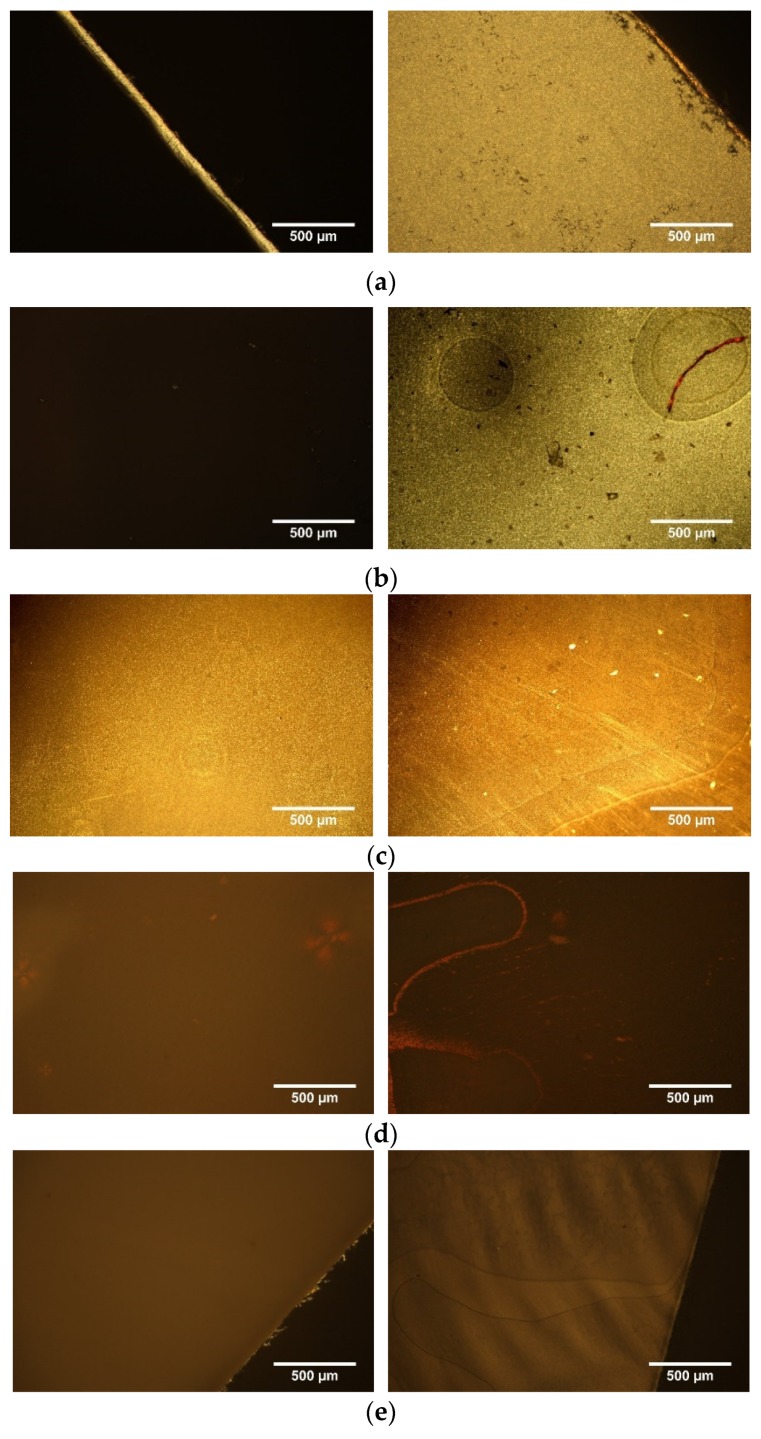
Optical textures of the created films, before (**left side**) and after annealing at 120 °C (**right side**) for l,d-PLA (**a**) and l,d-PLA:5CB in the ratio 10:1 *w*/*w* (**b**), 10:3 *w*/*w* (**c**), 10:5 *w*/*w* (**d**) and 10:10 *w*/*w* (**e**).

Textures registered at room temperature for created film of pure l,d-PLA and four compositions before heating and after annealing at 120 °C for a few minutes are presented in Figure 5. Films of pure l,d-PLA and composite 10:1 were optically isotropic at room temperature (amorphous phase) and show black image under the polarizing microscope with crossed polarizers. Therefore, only the edge of the created films, as an example for pure l,d-PLA, is presented in Figure 5. As it is seen, after annealing these films transformed to crystal phase which made them visible with crossed polarizers. Films with more 5CB admixtures were visible before and after annealing and they were changing with increasing the concentration of 5CB (Figure 5).

**Figure 6:**
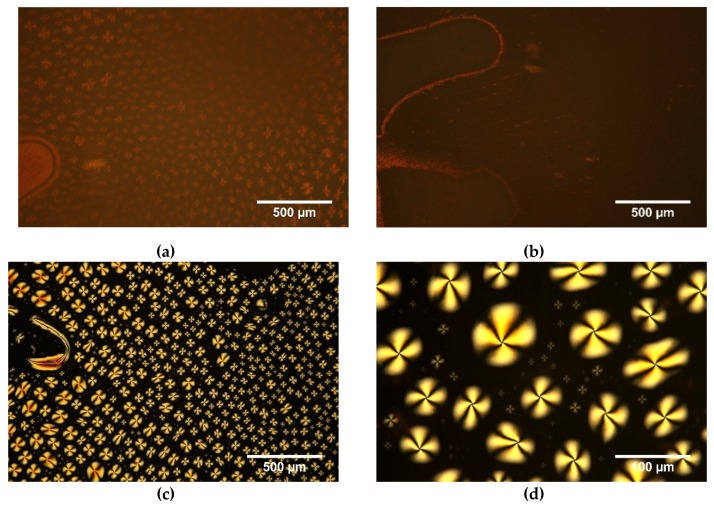
Optical textures after annealing and cooling down to room temperature with (**a**) and without (**b**) cover glass for composite 10:5 *w*/*w* and for 5CB on cover glass (**c**,**d**); crossed polarizers, room temperature.

It was noticed, that during annealing at 120 °C a part of 5CB was pushing out of the polymer matrix. Figure 6 presents textures registered for the l,d-PLA:5CB composite 10:5 *w*/*w* between two glass slides and without cover glass after annealing. All the observed anisotropy centres were placed on the cover glass because without the cover glass these centres are absent (Figure 6b). In turn, they were well visible on the cover glass (see Figure 6c,d). It suggests that 5CB was forced out of the l,d-PLA polymer matrix during cold crystallization. It seems that this is due to the very low viscosity of 5CB at 120 °C and its outflow from the rigid structure of the l,d-PLA polymer matrix, which means that heating to such a high temperature destroys the composite.

**Figure 7:**
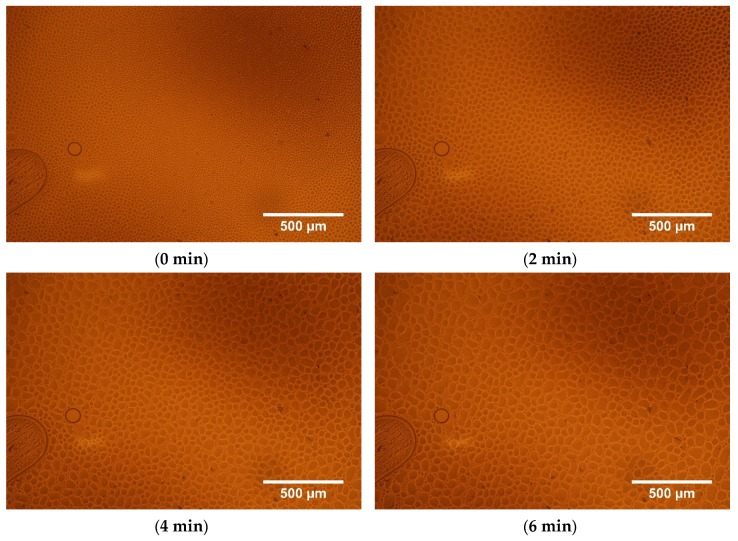
Optical textures (without analyser) of l,d-PLA:5CB composite with ratio 10:5 *w*/*w* registered at 120 °C versus time: process of pushing out the droplet of 5CB.

Process of pushing out the 5CB from the l,d-PLA polymer matrix is presented in Figure 7. As it can be observed, the higher content of 5CB is pushed out with time: the droplets are formed on cover glass and they are getting bigger. The results of analysis of the images by Fiji program are presented in Table 2.

One can noticed that the number of droplets decreases with time (from 9372 to 4980), the average size of one drop increases at the same time (from 190 to 450 μm^2^) and the total area occupied by 5CB increases (from 1.8 to 2.2 mm^2^). The circularity parameter is close to 1 what means that most of the droplet have a circular shape as it is well visible in Figure 6 and Figure 7.

**Figure 8:**
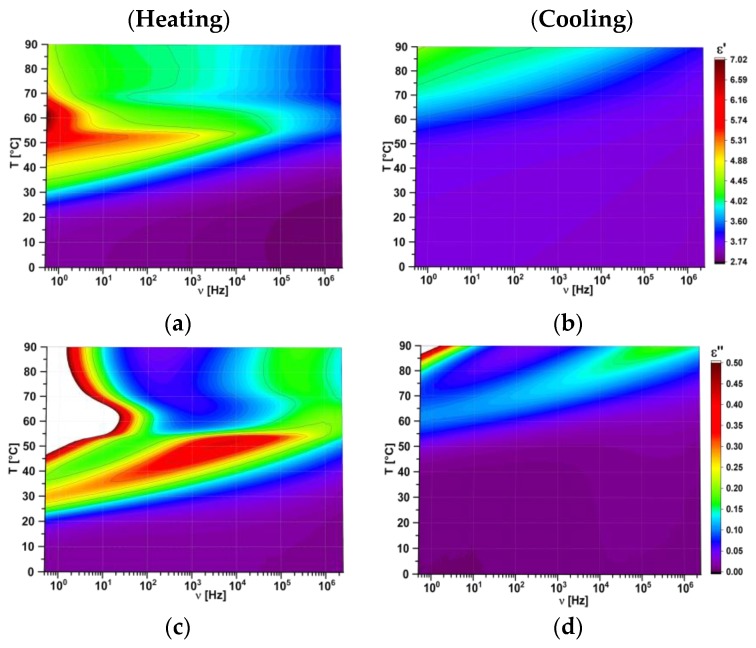
Three dimensional maps of real (**a**, **b**) and imaginary (**c**, **d**) part of dielectric permittivity for pure l,d-PLA: heating (**on the left**) and cooling (**on the right**).

FDDS method was used to study electric properties of created hybrid compositions. All the data are presented as 3D maps where colour indicates the value of studied parameter. As an example, the dielectric absorption and dispersion as 3D maps for pure l,d-PLA polymer film are presented in Figure 8.

**Figure 9:**
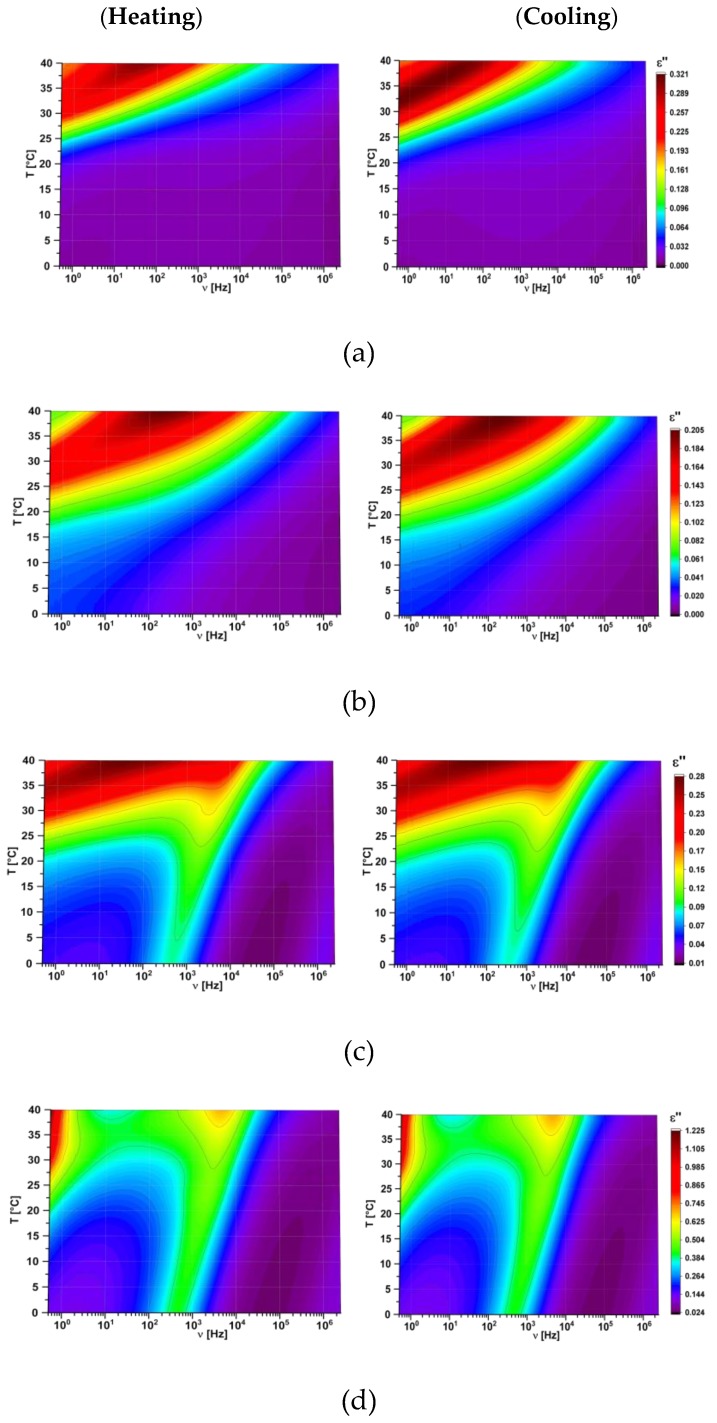

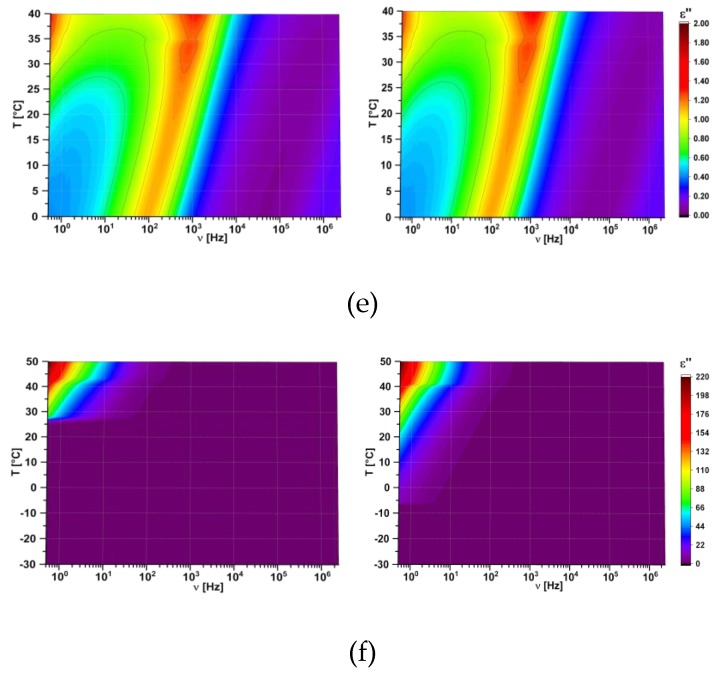
Three dimensional maps of imaginary part of dielectric permittivity for created films at heating (**on the left**) and cooling (**on the right**), in the temperature range 0 to 40 °C for l,d-PLA (**a**) and l,d-PLA:5CB in the ratio 10:1 *w*/*w* (**b**), 10:3 *w*/*w* (**c**), 10:5 *w*/*w* (**d**), 10:10 *w*/*w* (**e**) and 5CB (**f**).

For all the created hybrid films the maps for heating and cooling are different because the cold crystallization occurred at heating and the materials changed their physical state (as it was shown in Figure 4). Films in crystal form were much more inflexible than in amorphous form and for this reason the next research were performed in the temperature range from 0 °C to 40 °C. In this temperature range pure 5CB can exist in crystalline, nematic or isotropic phases (Figure 3b). It turned out that in this temperature range the maps for new created films obtained during heating and cooling are identical, as it is presented in Figure 9. It strongly suggests that created films are stable. In turn, the value of ε′ increases with increasing the 5CB admixture (see Figure 9).

**Figure 10:**
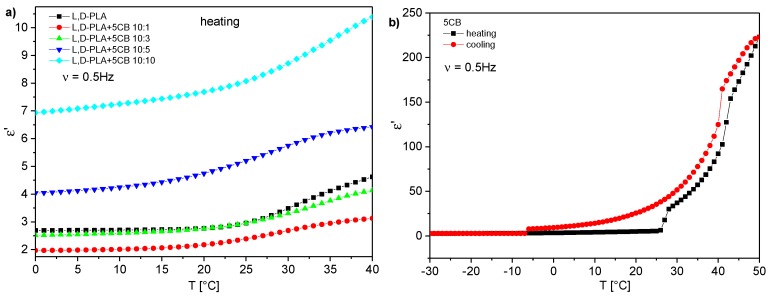
Dispersion at 0.5 Hz versus temperature for new created hybrid films during heating (**a**) and pure 5CB during heating and cooling (**b**).

Figure 10 presents temperature dependence of the dielectric constant (dielectric dispersion at 0.5 Hz) of created layers. The results for hybrid layers obtained at heating and cooling were identical therefore only results for cooling are presented in Figure 10a. Results for pure 5CB obtained during heating and cooling are different and both are presented in Figure 10b. The visible jumps both on heating and cooling occurred at temperatures associated with the phase transitions as one can compare with the DSC results presented in Figure 3b. On the other hand, in the case of hybrid layers the jumps were absent (Figure 10a) what is in good agreement with DSC result (Figure 3a) for this range of temperatures. Dielectric constant at room temperature is equal to 2.95 for pure l,d-PLA and composite 10:1 *w*/*w* and 2.39 for composite 10:3 *w*/*w* (see Figure 10a). For higher concentration of 5CB admixture it grows fast and is equal to 5.20 and 8.07 for composite 10:5 and 10:10, respectively. Taking into account that dielectric constant was calculated based on the thickness of the created layers one can conclude that as the 5CB admixture increases in l,d-PLA the dielectric constant is almost constant up to a concentration of 3:10 *w*/*w*, after which it fast increases. It is associated with a very high dielectric constant of 5CB (at cooling: 35.52 at 25 °C, Figure 10b), whose amount of dopant increases in composites, which increases the dielectric constant of the composites produced.

### 3.2. Mechanical Properties of l,d-PLA with 5CB and l,d-PLA:5CB:SWCN

Mechanical properties were investigated for created hybrid layers in order to assess:

(i) the influence of 5CB component and its amount on the properties of thin film;
(ii) select the ratio of l,d-PLA:5CB forming the thin film with the best mechanical properties;
(iii) the influence of SWCN on selected l,d-PLA:5CB composite with required mechanical properties on the properties of thin film.

**Figure 11:**
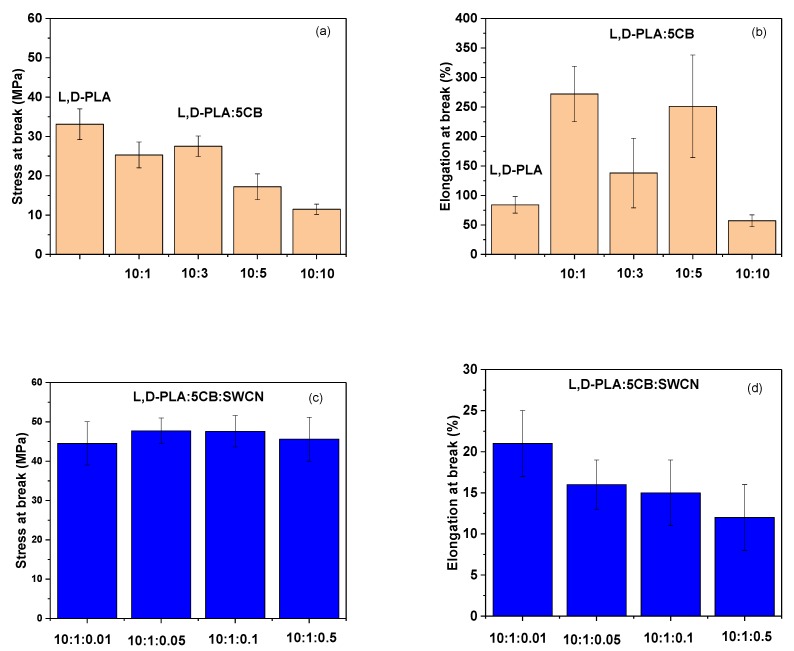
Stress at break and elongation at break of l,d-PLA:5CB (**a** and **b**, respectively) and l,d-PLA:5CB:SWCN (**c** and **d**, respectively).

All results are listed in Table 3. In the case of two-component samples there is a correlation between stress at break and the presence of 5CB compound for different samples (Figure 11a). Already 9% of the addition 5CB to the polymer matrix reduced the stress value at break up to one-third compared to pure polymer. At the same time the elasticity of thin film increased compared to starting material. A tendency was also found—increasing the amount of 5CB from 9% to 50% causes stiffening of the film, reducing the stress at break to 11.5 MPa and elongation at break to 57% of starting length (Figure 11b).

In general, the highest repeatability was observed for pure l,d-PLA and a mixture l,d,-PLA:5CB in which the polymer content was the highest (10:10 *w*/*w*). In other two cases, for mixture with 10:5 *w*/*w* and 10:1 *w*/*w* ratio of l,d-PLA:5CB, a clear strength limit was observed, that is, at high deformations of the sample normal stress increases, in some cases exceeding the yield point itself. The strength of the sample with ratio 10:10 *w*/*w* and 10:3 *w*/*w* after reaching the yield point, decreased.

The film composed of l,d-PLA:5CB (10:1 *w*/*w*) showed the highest elasticity, hence it has been selected as the base composition to add single-walled carbon nanotubes. Our previous study revealed that adding even a small amount of SWCN reduces the stress value due to loss of the elasticity [27]. Therefore, high elasticity is a desired property for the starting polymeric matrix.

In order to evaluate the effect of adding SWCN into polymeric matrix composed of l,d-PLA:5CB (10:1 *w*/*w*) four samples containing addition of 0.01, 0.05, 0.1 and 0.5 part by weight of carbon nanotubes were tested. The results are summarized in Table 3. Generally, the highest mean breaking stress was noted for samples l,d-PLA:5CB:SWCN (10:1:0.1 *w*/*w*/*w*) and l,d-PLA:5CB:SWCN (10:1:0.05 *w*/*w*/*w*) with values 47.6 MPa and 47.7 MPa, respectively. On the other hand, the lowest value was obtained for sample l,d-PLA:5CB:SWCN (10:1:0.01 *w*/*w*/*w*), giving value 44.5 MPa. However, taking into account the experimental error the values are oscillating within very similar range (Figure 11c). Evaluating the elongation at break, a gradual decrease of values on increasing carbon nanotubes content, starting from 21% for l,d-PLA:5CB:SWCN (10:1:0.01 *w*/*w*/*w*) and ending with 12% for l,d-PLA:5CB:SWCN (10:1:0.5 *w*/*w*/*w*) was observed (Figure 11d). In comparison to our previous report [27] where we studied films made only of l,d-PLA and SWCN, in this work we discovered that the addition of 5CB to l,d-PLA:SWCN matrix demonstrated the reverse of some mechanical properties. Namely, the stress at break values decreases and elongation at break remain at similar level with addition of carbon nanomaterial to pure polymer. In current study, the opposite trends can be explained by the influence of 5CB on formation of film internal structure, as it was observed in studies for polycarbonate, where 5CB was used as plasticizer [59].

### 3.3. Thermal Images of l,d-PLA with 5CB and SWCN

**Figure 12:**
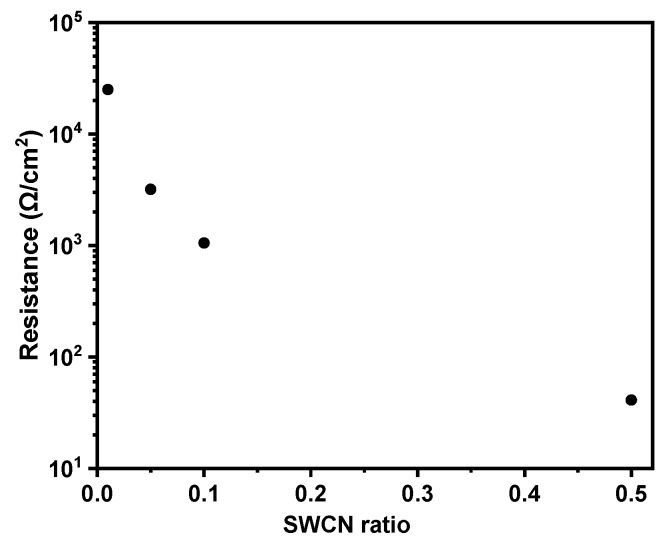
The correlation of resistance versus SWCN ratio for composite materials containing l,d-PLA, 5CB and SWCN.

Thermal imaging technique coupled with current registration was used to observe for overall behaviour of composite materials while an external potential was applied. As it was described in mechanical studies the film composition l,d-PLA to 5CB in 10:1 *w*/*w* ratio possessed the best mechanical properties, however due to its insulating properties it was doped with carbon nanotubes in order to give conductive properties. In general, samples based only on l,d-PLA and 5CB in different rations are good insulators with resistance in the range of mega ohms in both configurations in- and through-plane. Therefore, four different contents of SWCN in polymeric composite film were tested. The electrical conductivity measurements at in-plane configuration revealed that even a small amount of carbon nanotubes enables current flow, giving the average resistance values equal to 25,061.4 Ω, 3186.8 Ω, 1051.5 Ω and 41.0 Ω, for 0.01, 0.05, 0.1 and 0.5 weight portion of SWCN, respectively. In the experimental setup the distance between external connections was 1.3 cm. As it can be seen in Figure 12 the correlation of resistance versus SWCN content changes exponentially, decreasing the resistance for higher carbon nanotube content, as expected. All samples were stable in all measured potential range.

**Figure 13:**
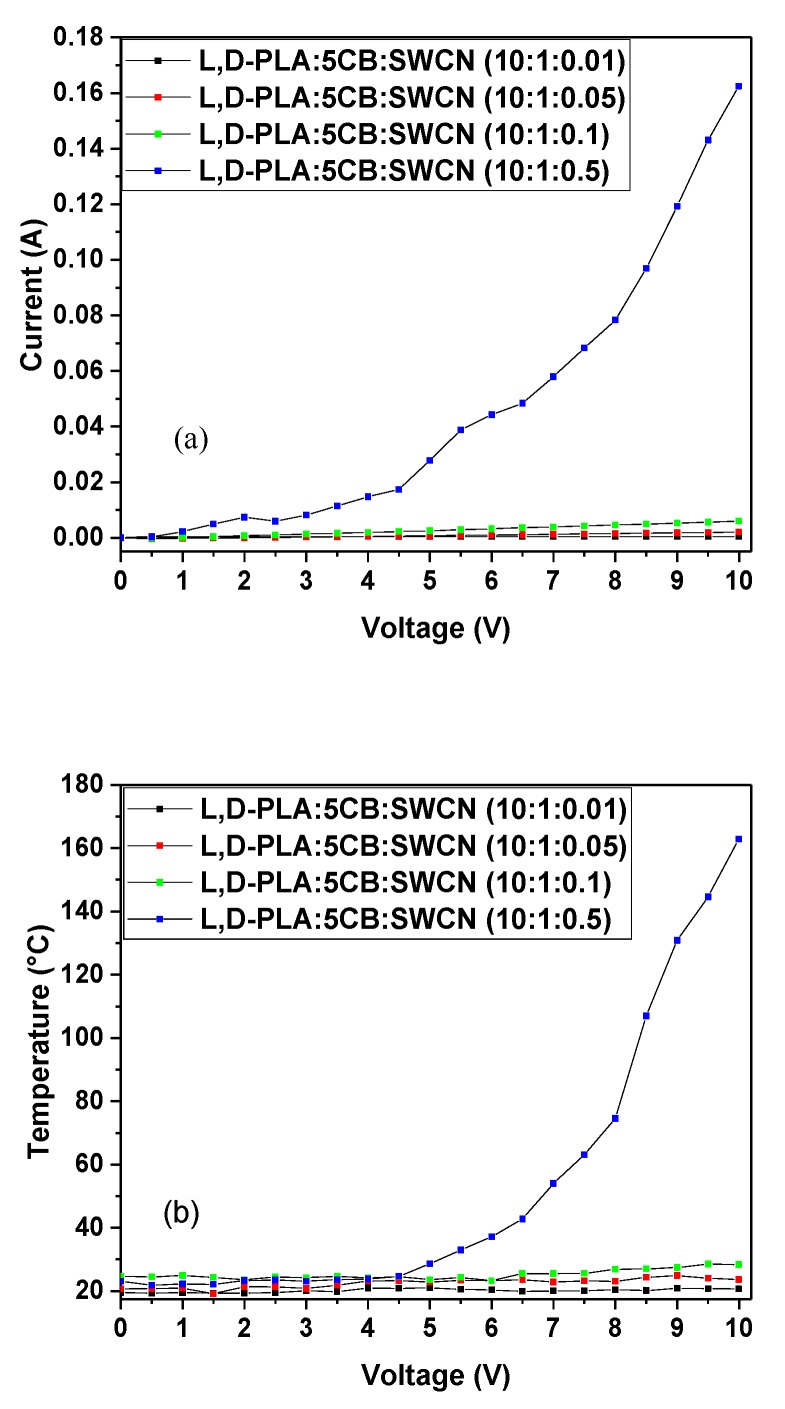
The correlation of (**a**) current versus applied potential and (**b**) temperature versus applied potential for composite materials containing l,d-PLA, 5CB and SWCN.

The analysis of the current-voltage curve demonstrated a linear increase of current only in the case of samples with high resistance of kΩ (Figure 13a). For the highest content of SWCN the current values incremented linearly at two different slopes connected by a deflection between 5 and 7 V. After 7 V the curve became steeper. It might be related to some phase transition in the sample. The carbon nanotubes in polymer matrix could rearrange according to external stimulus, like electric field improving conductivity [34].

**Figure 14:**
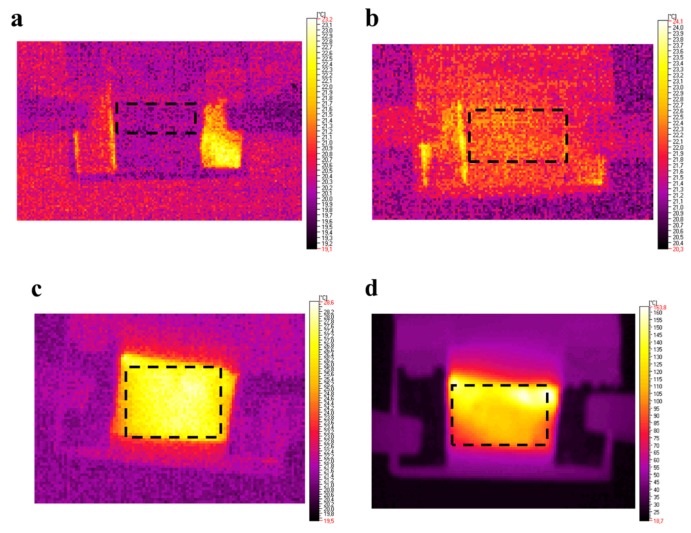
Thermographic images of films with different content of l,d-PLA:5CB:SWCN with respective ratios of 10:1:0.01 *w*/*w*/*w* (**a**), 10:1:0.05 *w*/*w*/*w* (**b**), 10:1:0.1 *w*/*w*/*w* (**c**) and 10:1:0.5 *w*/*w*/*w* (**d**) for applied potential equal to 10 V. In black dashed frame an area used for thermal study has been marked.

The analysis of thermal response to external potential showed that for low conductive samples containing 0.01, 0.05 and 0.1 of SWCN weight portion, the highest observed temperature did not exceed 30 °C (Figure 13b). Whereas, the composite with composition l,d-PLA:5CB:SWCN (10:1:0.5 *w*/*w*/*w*) demonstrated an logarithmically increasing temperature, reaching slightly above 160 °C for potential of 10 V. The deflection of the curve was observed in the temperature range corresponding to glass transition for pure polymeric matrix as observed in DSC study. It was noticed that the higher the resistance, the lower temperature was observed, suggesting the pure conductive nature of samples. The analysis of thermographs revealed that the films heat up rather homogenously without any detectible defects, as it can be seen in Figure 14 for all samples studied.

## 4. Conclusions

In this work, polymer compositions based on biodegradable l,d-PLA, liquid crystal 5CB and SWCN were fabricated and investigated by thermal, electrical and mechanical studies. Following main conclusions can be drawn:

The addition of 5CB to l,d-PLA increased the flexibility of polymeric base films and a strength limit, as evidenced by the nature of the rupture of the l,d-PLA samples compared to two-component films;

The best material flexibility and short-term strength were obtained for l,d-PLA sample with 9% 5CB content (10:1 *w*/*w* ratio), which means that a small addition of 5CB has a beneficial effect on the mechanical properties of the thin film. Increasing the proportion of this component above 30% reduces the strength and elasticity of the film;

The addition of SWCN to the base polymeric matrix with liquid crystal 5CB (10:1 *w*/*w*) caused an almost twofold increase in the strength of the tested materials and an almost tenfold decrease in their elasticity;

Addition of SWCN into polymeric matrix containing 5CB gave conductive nature to the thin films. The best electrical conductivity was observed for l,d-PLA:5CB:SWCN with ratio 10:1:0.5 *w*/*w*/*w*, showing an average resistance of 41.0 Ω and thermal response up to 160 °C without causing any damages;

Thermal effect accompanying the passing current could have positive influence on the registered current flow, possibly causing additional rearrangement of SWCN in the thin film after reaching the glass transition temperature of l,d-PLA:5CB (10:1 *w*/*w*);

Only binary hybrid layers are dielectric and the dielectric dispersion ε’ increases with increasing the 5CB admixture except the hybrid layer with the lowest concentration of 5CB (10:1) where dispersion is even smaller than for the layer made of pure l,d-PLA;

Films of pure l,d-PLA and composites with low admixture of 5CB are optically isotropic at room temperature (amorphous phase) while with more 5CB admixture become anisotropic. After annealing all created binary layers are optically anisotropic and they are changing with increasing the concentration of 5CB.

## Figures and Tables

**Table 1:** Transition temperature of created l,d-PLA:5CB films in various ratio of 5CB and for l,d-PLA:5CB:SWCN in various ratio of SWCN based on DSC method.

Compositions [*w*/*w*/*w*]	Glass Transition [°C]	Transition to Liquid [°C]
l,d-PLA	61	161
l,d-PLA:5CB, 10:1	45	152
l,d-PLA:5CB, 10:3	37	148
l,d-PLA:5CB, 10:5	31	147
l,d-PLA:5CB, 10:10	30	141
l,d-PLA:5CB:SWCN (10:1:0.01)	52	155
l,d-PLA:5CB:SWCN (10:1:0.05)	48	154
l,d-PLA:5CB:SWCN (10:1:0.1)	50	155
l,d-PLA:5CB:SWCN (10:1:0.5)	53	155

**Table 2:** Parameters of shape for analysed images.

Time [min]	Amount of Droplets	Total Area of Droplets [μm^2^]	Average Size [μm^2^]	Area Occupied by Droplets [%]	Circularity
**0**	9372	1,784,464	190	55	0.74
**1**	7588	1,837,159	242	57	0.76
**2**	6653	1,997,143	300	62	0.76
**4**	5600	2,175,919	389	67	0.76
**6**	4980	2,243,426	450	69	0.76

**Table 3:** Tensile test performance for two- and three-component hybrid layers.

Composition (*w*/*w*)	Thickness (mm)	Cross-Section Area (mm^2^)	Length (mm)	Stress at Break (MPa)	Elongation at Break (%)
l,d-PLA	0.080 ± 0.005	0.89	8.2	33.1 ± 3.9	84 ± 14
l,d-PLA:5CB (10:1)	0.089 ± 0.004	1.10	9.4	25.3 ± 3.3	272 ± 47
l,d-PLA:5CB (10:3)	0.048 ± 0.004	0.60	10.0	27.5 ± 2.6	138 ± 59
l,d-PLA:5CB (10:5)	0.057 ± 0.010	0.69	9.2	17.2 ± 3.3	251 ± 87
l,d-PLA:5CB (10:10)	0.072 ± 0.004	0.86	10.6	11.5 ± 1.3	57 ± 10
l,d-PLA:5CB:SWCN (10:1:0.01)	0.043 ± 0.002	0.50	9.7	44.5 ± 5.5	21 ± 4
l,d-PLA:5CB:SWCN (10:1:0.05)	0.047 ± 0.001	0.60	8.5	47.7 ± 3.3	16 ± 3
l,d-PLA:5CB:SWCN (10:1:0.1)	0.055 ± 0.002	0.70	9.7	47.6 ± 4.0	15 ± 4
l,d-PLA:5CB:SWCN (10:1:0.5)	0.050 ± 0.002	0.60	9.7	45.6 ± 5.6	12 ± 4
